# Efficacy and safety of immune checkpoint blockade for brain metastases

**DOI:** 10.2217/cns-2018-0018

**Published:** 2019-03-11

**Authors:** Maya Harary, David A Reardon, J Bryan Iorgulescu

**Affiliations:** 1 Harvard Medical School, Boston, MA, 02215, USA; 2 Department of Neurosurgery, Computational Neuroscience Outcomes Center, Brigham & Women's Hospital, Boston, MA, 02215, USA; 3 Department of Medical Oncology, Center for Neuro-Oncology, Dana-Farber Cancer Center, Boston, MA, 02215, USA; 4 Department of Pathology, Brigham & Women's Hospital, Boston, MA, 02215, USA; 5 Department of Medical Oncology, Dana–Farber Cancer Institute, Boston, MA, 02215, USA

**Keywords:** brain metastasis, CTLA-4, immune checkpoint blockade, immunotherapy, PD-1, PD-L1

As underscored by the 2018 Nobel Prize in Physiology for Medicine, immune checkpoint blockade (ICB) has revolutionized the management for a spectrum of advanced cancer types, both in the subsequent-line as well as first-line therapy settings. Since 2011, seven different agents targeting three different immune checkpoints have been approved by the US FDA. These include monoclonal antibodies against CTLA-4 (i.e., ipilimumab), PD-1 (i.e., nivolumab, pembrolizumab, and cemiplimab), and PD-L1 (i.e., atezolizumab, avelumab, and durvalumab); now with FDA-approved indications for advanced and/or unresectable melanoma, non-small-cell lung carcinoma (NSCLC), head and neck and cutaneous squamous cell carcinoma, renal cell carcinoma (RCC), urothelial carcinoma, classical Hodgkin lymphoma, hepatocellular carcinoma, gastroesophageal adenocarcinoma, Merkel cell carcinoma, cervical adenocarcinoma, primary mediastinal B-cell lymphoma, and solid cancers with mismatch repair deficiency or high microsatellite instability. However, most of the initial ICB clinical trials had included disproportionately fewer patients with brain metastases (BMs) despite the increasing predilection for CNS metastases displayed by many cancer types. This was in part due to a number of challenges that face therapeutic trials for BMs, including concerns about: the blood–brain barrier (BBB) permeability of therapeutics; the potentially confounding effects of other treatment modalities often necessary for BMs (e.g., whole-brain RT, stereotactic radiosurgery [SRS], or surgical resection), and that therapeutic efficacy might be diminished by the high-dose corticosteroids that are commonly needed to treat symptomatic cerebral edema. Historically, CNS metastases have proven particularly challenging to treat, with most therapeutic approaches providing minimal clinical benefit for patients – highlighting the need to explore the intracranial efficacy of novel new therapeutic modalities like immune checkpoint immunotherapy.

## ICB for cancer

By co-opting various inhibitory immune checkpoint pathways, multiple cancers have demonstrated a proficiency for evading native antitumoral immune activity. ICB agents can block these exploited immune checkpoint pathways and, consequently, promote the expansion of functional tumor-specific T cell responses [1]. The first approved ICB, the monoclonal antibody ipilimumab, blocks the immunosuppressive CTLA-4 protein on T cells, thereby permitting the priming of T cells during their activation phase. Subsequently, nivolumab, pembrolizumab, and cemiplimab were approved – monoclonal antibodies against PD-1 that block binding with PD-1's ligands (PD-L1 and PD-L2) and thus uninhibiting T cells during their effector phase. The third group of approved ICBs (e.g., atezolizumab, avelumab, and durvalumab) also blocks the interaction of PD-1 with its ligands, but by targeting PD-L1 instead. The initial clinical trials of ICBs focused on unresectable/metastatic melanoma, demonstrating dramatic successes that led to the median overall survival (OS) of Stage IV melanoma patients more than doubling nationwide [2]. However, even though 3–9% of patients in the pioneering KEYNOTE-001 (NCT01295827), KEYNOTE-006 (NCT01866319), CheckMate-066 (NCT01721772), CheckMate-067 (NCT01844505), and CheckMate-069 (NCT01927419) clinical trials had metastatic brain involvement, the intracranial response rates were not specifically reported.

## ICB monotherapy for BMs

In the following years since, the findings from a number of retrospective series and clinical trials focusing exclusively on ICBs in BMs have been reported – largely for patients with melanoma BMs, but also for cohorts of NSCLC or RCC BMs. Long considered an immune-privileged sanctuary for cancers, the results from these initial studies challenged conventional dogma by demonstrating the intracranial activity of ICBs. Across all of these diverse initial single-agent ICB studies, the median time from ICB treatment to intracranial response was approximately 2 months and the dosing regimens were relatively well tolerated. The first such trial evaluated single-agent anti-CTLA-4 ipilimumab (10 mg/kg given every 3 weeks for four doses, followed by 10 mg/kg every 12 weeks) in melanoma BM patients (NCT00623766) [3]. Among 51 neurologically asymptomatic melanoma BM patients off corticosteroids, 24% showed intracranial disease control at 12 weeks, with a median OS of 7.0 months. Among 21 symptomatic patients on corticosteroids, the intracranial disease control rate fell to 10%, with a corresponding median OS of only 3.7 months. By adding fotemustine (a nitrosourea alkylating chemotherapy with BBB-penetrability) to single-agent ipilimumab, the intracranial disease control for asymptomatic melanoma BMs (n = 20) improved to 50% at 24 weeks and the median OS improved to 12.7 months (NCT01654692, i.e., NIBIT-M1) [4]. Notably, 39% of patients were alive at 2 years and 71% of these patients had received no prior BM therapy.

In contrast to anti-CTLA-4 monotherapy, the use of anti-PD-1 pembrolizumab monotherapy (10 mg/kg every 2 weeks for up to 2 years, followed by 2 mg/kg every 3 weeks) for asymptomatic melanoma BMs (n = 23) was associated with a 2-year OS rate of 48% and median OS of 17.0 months (NCT02085070) [5]. Of 15 patients with evaluable intracranial responses, 27% had complete responses and 13% had partial responses, all which persisted at least 2 years. Pembrolizumab was well tolerated, with 20% of patients experiencing Grade III adverse effects (AEs, including 6% that were neurological), while the most common neurologic treatment-related AEs were Grade I and II gait disturbances or headaches. Additionally included in this single-agent pembrolizumab trial were 18 patients with asymptomatic NSCLC BMs, of whom 33% demonstrated intracranial objective disease responses (including 22% with complete responses), while 24% experienced Grade III or IV treatment-related AEs (none of which neurological) [6]. For NSCLC BM patients, nivolumab monotherapy (3 mg/kg every 2 weeks) also achieved adequate safety and encouraging durability of responses based on two reports. In the first, among five asymptomatic patients there were no Grade III or IV treatment-related AEs and 40% demonstrated intracranial responses (one complete and one partial response) that persisted at least 24 weeks [7]. Neurological AEs were present in 44% of patients, mostly manifesting as Grade I and II headaches. In a larger trial of 43 heavily pretreated NSCLC BM patients, nivolumab was associated with a 51% intracranial disease control rate after a median follow-up of 5.7 months, including 9% with an intracranial objective response rate, but the median OS was only 7.5 months [8–10]. In this trial, only 11% of patients experienced neurological AEs, but half of which were Grade III or IV. Nivolumab monotherapy (3 mg/kg every 2 weeks) outcomes have also been preliminarily reported for: 16 melanoma BM patients that were symptomatic or failed local BM therapy, in which only 6% showed intracranial response, 81% had progressive disease, and median OS was 5.1 months (NCT02374242: anti-PD1 brain collaboration [ABC]); 32 asymptomatic RCC BM patients (but were permitted corticosteroids) in which 19% showed intracranial response, 41% had progressive disease, and the 1-year OS was 67% – outcomes comparable to the study's non-BM metastatic RCCs; and 64 evaluable asymptomatic RCC BM patients, in which 17% showed intracranial response, 39% had progressive disease, and 1-year OS was 62% (NCT03013335) [9–11].

Only preliminary intracranial data for anti-PD-L1 single-agent ICBs have been reported. Among 38 asymptomatic NSCLC BM patients previously treated with cytotoxic chemotherapy, atezolizumab (1200 mg every 3 weeks) was associated with a median OS of 20.1 months, compared with only 11.9 months with docetaxel (n = 42, 75 mg/m^2^ every 3 weeks; NCT02008227, i.e., OAK trial) [12]. When pooled with an additional 41 NSCLC BM patients from four ongoing atezolizumab trials (NCT01375842: PCD4989g, NCT02031458: BIRCH, NCT01846416: FIR, and NCT01903993: POPLAR), safety was comparable between patients with and without BMs: 9% of BM patients had treatment-related serious AEs (compared with 10%), of which none were neurological (compared with 0.5%), while 10% of patients had to discontinue ICB treatment due to AEs (compared with 7% in the comparably treated non-BM patients) [13].

## ICB combination therapy for BMs

By modulating different components of antitumoral T cell responses, combined blockade of CTLA-4 and PD-1 signaling pathways has demonstrated additive clinical benefit in ICB trials. In the past year, the results from two much-anticipated Phase II trials of combination ICB for BMs have been unveiled, although these have only been aimed at melanoma BMs. Both employed the FDA-approved first-line dosing regimen of 1 mg/kg nivolumab plus 3 mg/kg ipilimumab every 3 weeks for four doses, followed by 3 mg/kg nivolumab every 2 weeks. Of the 35 (NCT02374242: anti-PD1 brain collaboration [ABC]) and 94 (NCT02320058: CheckMate-204) evaluable asymptomatic melanoma BM patients, combination ICB showed an overall intracranial response rate of 46–55% (median follow-up of 14.0–17.0 months), including 17–26% with complete responses, a progressive disease rate of 33–46%, and estimated 2-year OS rate of approximately 50–70% [11,14]. Neither trial had yet reached a median intracranial progression-free survival. These outcomes compare favorably to the results from single-arm trials of ICB monotherapy for asymptomatic melanoma BMs including a nivolumab-only arm (n = 25; 3 mg/kg every 2 weeks), in which the overall intracranial response rate was only 20% (including 12% with complete responses), the progressive disease rate was 76% (median intracranial progression-free survival of 2.5 months), and an estimated 2-year OS rate approached 43%. As seen with ICB monotherapy, the intracranial responses of nivolumab plus ipilimumab were largely concordant with their extracranial responses and the responses developed rapidly (median 2.3 months from treatment).

In terms of safety, 96–97% of nivolumab plus ipilimumab patients experienced a treatment-related AE, of which 54–55% were Grade III or IV, including 6–7% that were neurological. The majority of these AEs were manageable, but 26–27% of patients required discontinuation of combinatorial ICB. Only one patient (out of the combined 129) experienced a Grade V treatment-related AE, due to myocarditis. By contrast, only 68% of the nivolumab-only cohort developed a treatment-related AE, of which 16% were Grade III (none of which were neurological, and no Grade IV) and only 4% discontinued therapy due to AEs. Based on their safety and durability of intracranial responses, together these two trials strongly support the use of nivolumab plus ipilimumab in the first-line setting for asymptomatic melanoma BMs. Of note, the intracranial responses and survival outcomes were somewhat blunted in patients that had previously failed BRAF and/or MEK inhibitors, in line with preclinical studies suggesting that immunoresistant phenotypes may arise as part of resistance to molecularly targeted inhibitors. These findings have been confirmed in ‘real-life’ national melanoma BM cohorts, in which ICB has been associated with more than doubling the median and 4-year OS of patients [15]. Combination ICB clinical trials specifically for BMs are now also underway for NSCLC (NCT02696993, NCT01454102).

## Novel combination approaches with ICB for BMs

Ongoing trials are investigating the prospect of combining ICB with antiangiogenic bevacizumab for symptomatic BMs in the hope that the antipermeability effect of bevacizumab may decrease cerebral edema and lessen the likelihood of corticosteroid use. Additionally, there has been growing excitement for the potential synergy between ICB and radiotherapy for BMs, due to RT's promising capacity to effect changes at distant sites beyond those that are irradiated (i.e., abscopal effect), to promote BBB permeability, to encourage tumor antigen release, and to stimulate pro-inflammatory cytokine release. Current National Cancer Comprehensive Network Guidelines recommend surgical resection of BMs followed by SRS of oligometastatic BMs (i.e., 1–3) to help establish local disease control; with whole-brain RT often reserved for more numerous BMs, local failure after SRS, or leptomeningeal involvement [16]. In an international meta-analysis of 534 melanoma, NSCLC, and RCC BM patients, concurrent ICB, and SRS (median 20 Gy in one fraction) demonstrated an improved 1-year OS rate of 65%, as compared with 52% in cases treated with nonconcurrent ICB and SRS. [17] When including hypofractionated stereotactic RT with SRS in a study of 260 BM patients, concurrent ICB continued to show improved OS (median 24.7 months) compared with nonconcurrent ICB and RT (14.5 months) and RT-alone (12.9 months); without a difference in the rates of radionecrosis (3%) or acute neurotoxicity [18]. As a result, multiple prospective trials have been initiated to better understand the role of combining different RT schema with ICB.

## Next steps for ICB therapies & BMs

As with extracranial metastases, there remains a pressing need for new biomarkers that effectively predict response to ICBs in BM patients. Increasingly being recognized is the risk for acquired immunoresistance and relapse in patients that initially respond to ICB, highlighting the need for multimodal therapeutic strategies that incorporate complementary immunotherapeutic approaches (e.g., novel ICBs, antigen-specific peptide vaccinations, oncolytic virotherapies, and/or adoptive cell therapies) [19]. The complex interactions between ICBs and molecularly targeted small molecule inhibitors in these patients are also an ongoing and critical area of investigation. Novel trial design is further needed to ensure that BMs are appropriately incorporated and that intracranial responses are rigorously evaluated. Nevertheless, these preliminary results from the aforementioned BM studies and clinical trials have confirmed the benefits of ICBs and defined new strategies for the caring of patients with a spectrum of BM types.
